# High‐Resolution Computed Tomography Imaging of the Cranial Arterial System and Rete Mirabile of the Cat (*Felis catus*)

**DOI:** 10.1002/ar.24251

**Published:** 2019-10-01

**Authors:** E. Leon Kier, Gerald J. Conlogue, Zhenwu Zhuang

**Affiliations:** ^1^ Yale University School of Medicine New Haven Connecticut; ^2^ Quinnipiac University Hamden Connecticut; ^3^ Yale University School of Medicine New Haven Connecticut

**Keywords:** cat, *Felis catus*, multiplanar high‐resolution micro‐computed tomography (CT), cerebral arteries, orbital arteries, facial arteries, rete mirabile, carotid rete, extracranial rete, skull bones, lacrimal canal, infraorbital canal, sphenopalatine foramen

## Abstract

The objective of this study was to investigate the possibility of obtaining high‐resolution multiplanar computed tomography (CT) imaging of the cranial arterial circulation of the cat (*Felis catus*), the rete mirabile, and components of the skull, utilizing preserved cat specimens with an arterial system that was injected with a radiopaque contrast compound in the early 1970s. Review of the literature shows no high‐resolution CT studies of the cat's cranial circulation, with only few plain radiographic studies, all with limited cranial vascular visualization. In view of the inability of the radiographic techniques available from 1970s to mid‐2000s to provide high‐resolution imaging of the arterial circulation within the intact skull and brain of the cat, without dissection and histologic sectioning and disruption of tissues, no further imaging was performed for many years. In 2010, a high‐resolution micro CT scanner became available, large enough to scan the entire nondissected head of the arterially injected cats. All the obtained CT images were processed with a software program that provided 3D volume rendering and multiplanar reconstruction with the ability to change the plane angulation and slab thickness. These technical features permitted more precise identification of specific arterial and bony anatomy. The obtained images demonstrated, with a nondestructive method, high‐resolution vascular anatomy of the cerebral, orbital, facial arterial system, the rete mirabile, and skull bone components of the cat, with details not previously described in the literature. Anat Rec, 302:1958–1967, 2019. © 2019 The Authors. *The Anatomical Record* published by Wiley Periodicals, Inc. on behalf of American Association of Anatomists.

Review of the literature reveals few radiographic studies of the cat's cranial circulation during the 1950s through the 1970s. These presented limited detailed vascular visualization (Daniel et al., 1953; Kormano, 1967; du Boulay and Verity, 1973; Kumar et al., 1976). Since that period, the literature shows no high‐resolution computed tomography (CT) studies of the cat's cranial circulation. Although the bony anatomy of the cat skull has been described in dissection manuals (Walker, 1970; Chiasson and Booth, 1973), no previous descriptions of a CT study of the cat's skull anatomy were found.

Advances in CT and digital reconstruction technologies have resulted in a number of studies with images that demonstrated, with a nondestructive method, detailed anatomy of the cerebral, orbital, facial arterial system, the rete mirabile, and skull bone components of a number of different animals. These include the studies of the head circulation of the domestic goat *Capra hircus* (O'Brien and Bourke, 2015), the mouse deer, the Sri Lankan spotted chevrotain *Moschiola memmina*, an even‐toed ungulate (O'Brien, 2015), the *Iguana iguana* (Porter and Witmer, 2015), the postnatal growth patterns in the giraffe (*Giraffa camelopardalis*) (O'Brien et al., 2016), and the vascular patterns in the heads of crocodilians (*Alligator mississippiensis*) (Porter et al., 2016).

In the majority of all animals, including fishes, amphibians, reptiles, birds, and a number of mammals, including primates, the cerebral blood supply is mainly by the internal carotid artery. In the cat and a number of even‐toed angulates (Kiełtyka‐Kurc et al., 2015; O'Brien, 2015), the cerebral blood supply is provided from the maxillary artery, a branch of the external carotid artery, via a complex plexus called the rete mirabile (Gillilan and Markesbery, 1963). There is a very long history of extensive continuing interest in the anatomy and the role of the rete mirabile of the cat and other mammals, going back to Galen in the second century AD (Baker, 1972; de Gutterez‐Mahoney and Schechter, 1972; du Boulay et al., 1975; McFarland et al., 1979) and even earlier ascribed to Herophilus in the third century BC (de Gutterez‐Mahoney and Schechter, 1972; McFarland et al., 1979). The large past literature dealing with the rete mirabile has been extensively discussed in recent publications that focus on the various physiological roles of the rete mirabile, including water conservation through selective brain cooling mechanisms (O'Brien, 2015; Strauss et al., 2017; O'Brien, 2018a, 2018b). The large number of publications studying the rete mirabile in the cat likely relates to the fact that the cat was one of the common laboratory subjects (Gillilan and Markesbery, 1963).

Since the middle ages there has been general scientific agreement as to the presence or absence of a rete mirabile in various animals. However, there has been disagreement as to the presence of an internal rete in addition to the external one, in some animals. Major disagreement persisted for many years about the presence of a rete in humans (de Gutterez‐Mahoney and Schechter, 1972). The latter authors in their review state that the term human rete mirabile should not be used until the time it is demonstrated by gross anatomic and histologic studies.

The continuing interest in the rete mirabile appears to relate to its physiologic roles which have been postulated as relating to regulation of vasoconstriction, blood pressure, and brain‐cooling mechanisms and for water conservation. Experiments in goats (*Capra aegagrus hircus*) demonstrated that resistance in the rete mirabile decreases during induced hypertension and increases in induced hypotension, with the result that blood flow to the brain is facilitated in hypertension and limits cerebral arterial blood flow in hypotension (Diéguez et al., 1988). These results were not confirmed in another study that by using complex computational models, which simulated the resistance within the rete mirabile and also the known blood pressure in the giraffe, found that based on these models, the carotid rete in artiodactyls, the even‐toed angulates, does not have a mitigating effect on the blood pressure (O'Brien and Bourke, 2015).

Discussed elsewhere is the very successful evolution of the artiodactyls, which started around 55 million years ago and which became a very successful diverse group, while the diversity of perissodactyls, the odd‐toed angulates, has decreased (Mitchell and Lust, 2008). These authors propose that the success of the artiodactyls was due to the presence of the carotid rete, which permitted them to conserve water and energy in hot environments and conserve body temperature in cold conditions. Thus, the presence of the rete permitted the artiodactyls to spread from arctic to the desert and equatorial forests.

An opposing view attributes the evolutionary success of the artiodactyls to improved locomotion and the specialized ruminant gastrointestinal tract which is more efficient in processing food with moderate fiber content (Janis, 2009). This author points out that during certain geologic periods, the perissodactyls also inhabited extreme climatic conditions and that over long periods there has been significant extinctions of groups of both the artiodactyls and the perissodactyls. The author also mentions that the development of the rete may have occurred during a benign climatic period and that the great majority of modern artiodactyls was an explosive radiation of a single group.

There has been extensive research regarding the advantage for animals possessing a rete mirabile in cooling the skull base, in particular the hypothalamic region. Research showed that in animals without a rete mirabile, such as the rabbit and monkey, the cerebral arterial blood temperature at the circle of Willis was the same as in the aortic arch, while in animals with a rete mirabile, such as the cat and the sheep, the cerebral arterial blood temperature at the circle of Willis was lower as in the aortic arch (Hayward and Baker, 1969). This was the result of the cerebral arterial blood cooled by the countercurrent heat exchange with the cooler venous blood from the nasal mucosa delivered to the venous system bathing the rete mirabile.

A series of detailed physiologic studies in Norwegian reindeers (*Rangifer tarandus*), an even‐toed angulate, studied in detail the histologic makeup of the nasal mucosa and its venous circulation (Johnsen et al., 1985). The study showed a venous pathway from the nose via the dorsal nasal vein, the angular oculi vein, and ophthalmic veins to the cavernous sinus indicating a potential for cooling of the cavernous sinus. A follow‐up study using angiography in the Norwegian reindeers showed the angular oculi veins constricted during cold stress and dilated during heat stress which in conjunction with a constriction of the facial vein shunts the cold venous return from the nose directly to the cavernous sinus (Johnsen et al., 1987). Further investigation and analysis of the vascular control of cooling of the body and the brain in heat‐stressed reindeers showed that sympathetic muscular contraction of the angular occuli veins and relaxation of the facial veins resulted in cold venous blood results general body cooling. Dilatation of the angular occuli veins and constriction of the facial veins resulted in the cold venous blood shunted for selective brain cooling (Johnsen and Folkow, 1988).

Recent publications have extensively reviewed these topics, focusing on the various physiological roles of the retia, including water conservation through selective brain cooling mechanisms (O'Brien, 2015; Strauss et al., 2017; O'Brien, 2018a, 2018b).

For many years, there has been continuing research regarding the physiologic role of the rete mirabile, among them is the cooling of the hypothalamic region by venous blood flow from the nose and mouth (Baker, 1972; du Boulay et al., 1975). The possible role of the rete in the autoregulation of vasoconstriction and blood pressure in the cat and even‐toed ungulates has been reviewed (Daniel et al., 1953; du Boulay and Darling, 1975).

A review of the literature shows that the great majority of the publications depict the rete mirabile diagrammatically. Thus, a nondestructive method of high‐resolution micro CT imaging of the cranial arterial system circulation, the rete mirabile, and skull components of the cat could be a significant contribution to the visualization of the cat's cranial anatomy. This was accomplished with 3D volume images and multiple planar projections.

## MATERIALS AND METHODS

### Radiopaque Contrast Material

In the early 1970s, eight arterially injected cat heads were obtained from a study that investigated the microcirculation of the eye in the cat, using a specially prepared radiopaque barium–gelatin compound that was injected into the carotid arteries following euthanasia (Rothman et al., 1975). Three of these injected specimens were dissected as part of the material for studies of the evolutionary and embryologic changes of the brain, cerebral arterial system, and cerebral ventricular system (Kier, 1974, 1977).

The barium–gelatin contrast agent, developed in the mid‐1960s for use in specimen radiography, demonstrated excellent results (Conlogue et al., 1978). The basis for the preparation is Micropaque® that was produced by Demancy and Co. Ltd., London, England, is a fine particle (0.1–0.4 μ) barium sulfate powder. In arterial injections, the small particulate size permits the barium to reach arterioles. Mixed with water, Micropaque® frequently flocculated or fell out of suspension (Kormano, 1967). A similar issue resulted in its removal from clinical applications. For specimen radiography, in order to maintain the powder in suspension, mucilago acacia was added. Mucilago acacia was prepared by adding 1.0 g gum Arabic to 10 g supersaturated sugar solution. The gum Arabic and the supersaturated sugar solution were combined in a blender with 10 g of mucilago acacia, 15 g of gelatin, 150 g of the fine barium powder, and 200 mL of hot, but not boiling, water. To allow successful dissection, gelatin was added to the mixture. When protein, the basis of gelatin, is treated with a 10% buffered formalin, the protein remained solid, preventing the medium from returning to a liquid state. In addition, it remained within the structure, not only when dissected (Kier, 1974) but also when subjected to sectioning. The precise steps in preparing this contrast media are described elsewhere (Conlogue et al., 1978).

### Specimen Preparation and Injection

Specimen preparation is the most important factor for a successful injection. Whenever possible, arterial and venous injections should be done under fluoroscopy to determine when a sufficient volume has been delivered and to detect any possible extravasation of contrast medium. At the end of injection, cooling is the key. For small specimens, immersion in liquid nitrogen will instantly solidify the contrast media. However, refrigeration for 24 hr will provide sufficient cooling for small and large specimens. Once the cooling process has been achieved, the next stage is fixation of the gelatin component of the contrast media. This was achieved by immersing the specimens in 10% buffered formalin. To assure that the brain is immersed in formalin, a segment of the upper skull and dura were removed. The detailed steps in specimen preparation and contrast injection techniques are described elsewhere (Rothman et al., 1975; Conlogue et al., 1978). The cat specimens were kept in 10% buffered formalin until 1996 when the specimens were transferred to a solution of WARDSafeTM (Ward's Science, Rochester, NY), a specimen preservative solution.

### Multiplanar High‐Resolution CT Imaging

Due to the inability of the radiographic techniques available from the 1970s to the mid‐2000s to provide high‐resolution imaging of the arterial circulation within the intact skull and brain, without dissection and disruption of tissues, no further imaging of the injected cats was performed for many years. In 2010, a high‐resolution CT scanner became available at Yale University, with a bore large enough to scan the entire nondissected head of the arterially injected cats. Three of the eight arterially injected cat heads, injected with a radiopaque barium–gelatin contrast agent in the 1970, were scanned in this study.

To achieve detailed information of the cerebral arterial system, the cat specimens were scanned with an explore CT‐120™ (GE Healthcare Piscataway, NJ), a high‐resolution small‐bore scanner (bore diameter up to 75 mm). The system consists of a high‐power rotating anode X‐ray tube and large‐area digital CCD detector. Rapid scans are facilitated by a 5 kW pulsed high‐output X‐ray tube. Acquisition parameters were 90 kVp, 40 mA, 2 × 2 detector bin model, 16 msec exposures per projection, 200 gains, and 50 offset. Typically, a full‐image data set covered 360 degrees in 0.4 degree steps for a total of 900 projections. These 900 projections were saved as a DICOM (Digital Imaging and Communications in Medicine) data set. DICOM is a standard for handling, storing, printing, and transmitting medical imaging and other data. Total acquisition time for a single data set was less than 10 min. Projections were initially reconstructed with a modified Feldkamp cone‐beam algorithm (Feldkamp et al., 1989). Data sets were reconstructed as 3D images by Microview software (GE Healthcare Piscataway) with effective voxel sizes of 50 μm (final spatial resolution) (Cuomo et al., 2019). MicroView, a general‐purpose 2D and 3D image viewer, is particularly suited for microCT reading and writing of DICOM image data. Its features include 2D and 3D image viewing, support for many image formats, region of interest extraction tools, geometric primitives, region‐growing, histogram‐based, iso‐surface, and a graphics processing unit‐accelerated volume renderer.

### Processing the CT Data

The original DICOM data set was analyzed on an Apple laptop computer, using the OsiriX MD image processing software (Rosset et al., 2004). OsiriX MD is an image processing software dedicated to DICOM images and runs on an Apple OS X systems, providing post‐processing functions such as 3D volume rendering and multiplanar reconstruction, with the ability to change the plane angulation, slab thickness, and delete bony and soft tissue structures from the data set. These technical features permit more precise identification of specific arterial anatomy. The ability to remove the overlying bony skull and the intracranial soft tissues is a key feature in helping visualize the intracranial vasculature. Additional selective setting of the OsiriX processing software permits visualizing of both the arteries and bones. The ability to change the slab thickness in the multiplanar images facilitated the identification of specific arteries and skull components.

## RESULTS

### Vascular Anatomy

The anatomic structures identified in this investigation included the visualization in 3D renderings (Fig. 1A,B) and in multiple projections (Figs. 2, 3, 4, 5, 6, 7, 8, 9, 10, 11, 12) of a large number of the arterial branches of the cranial arterial system and components of various skull bones.

**Figure 1:**
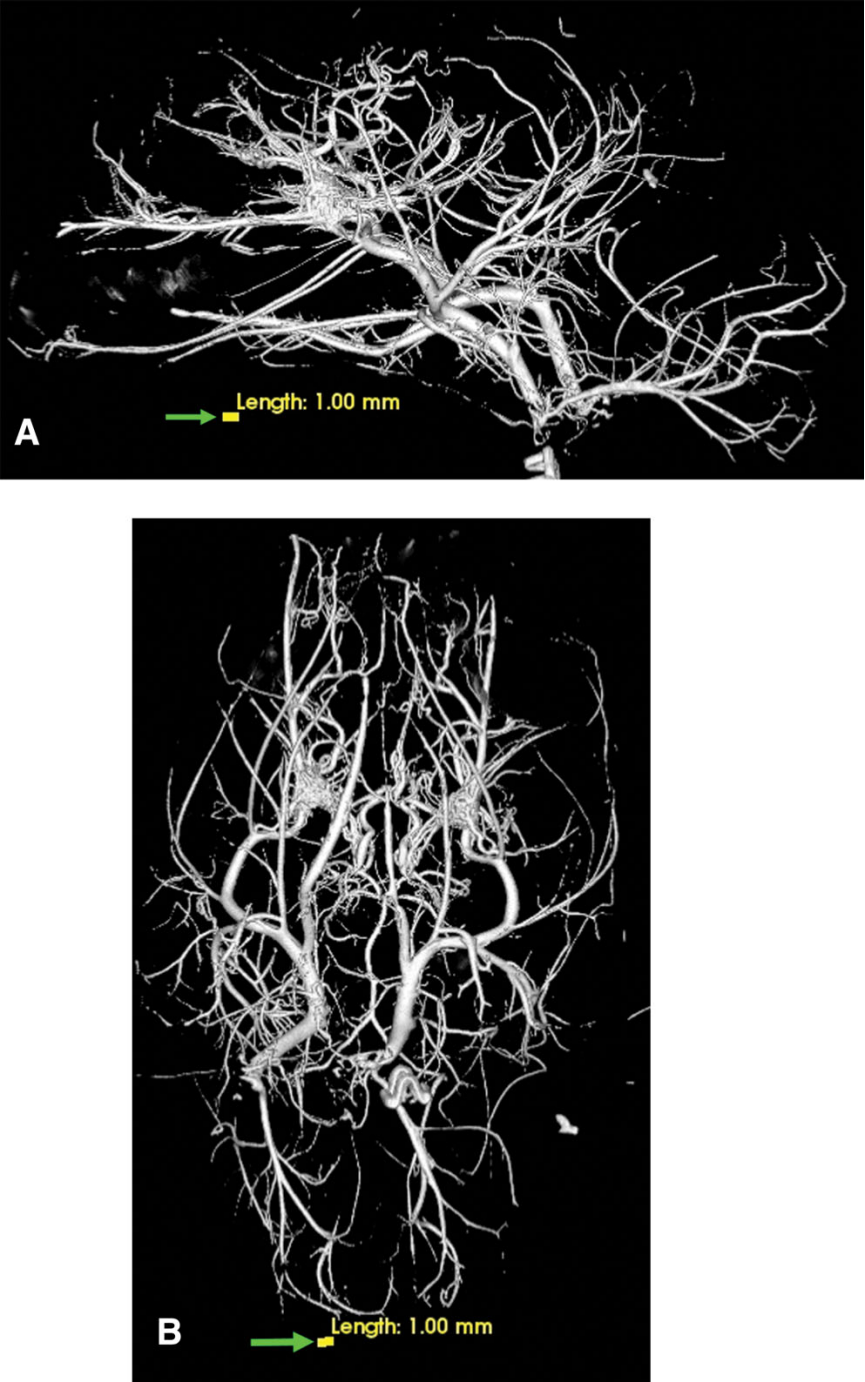
Lateral (**A**) and dorsoventral (**B**) 3D volume rendered projections of the cranial arterial circulation of a cat. Of note is the extent of the demonstrated vasculature in the bone and soft tissue free images and the small size of the vasculature when compared with the yellow colored 1.0‐mm scale bar (arrow). These 3D images can be rotated and turned in any direction or angle needed for the identification of a specific blood vessel.

**Figure 2:**
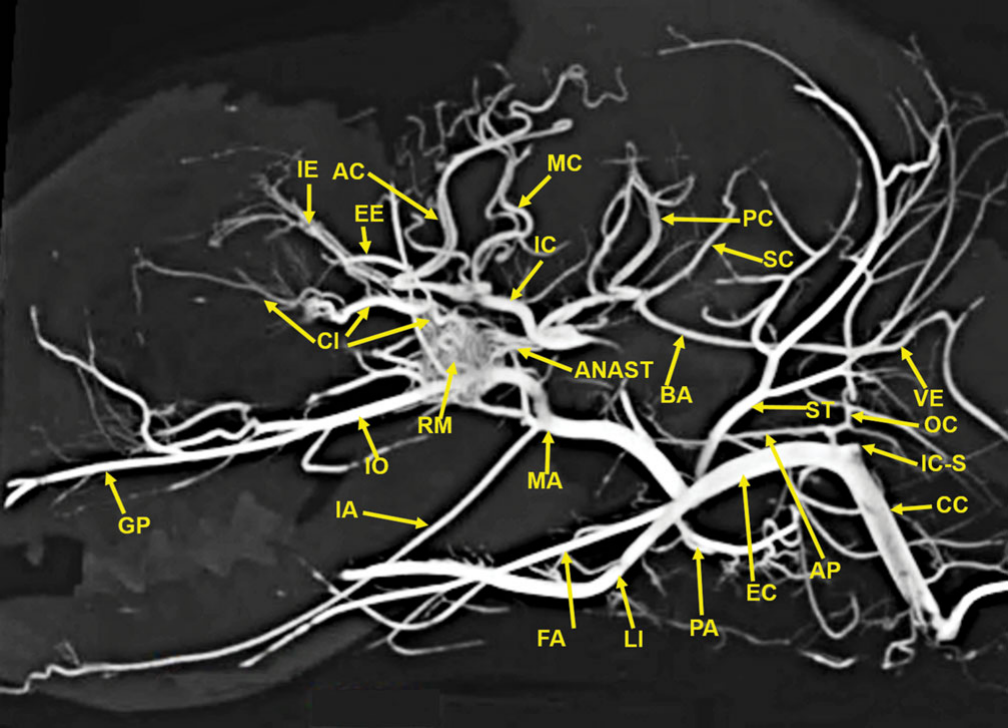
A 25‐mm‐thick lateral multiplanar reconstruction of the cranial arterial circulation of the cat, using maximum intensity projection. A large number of cranial arteries can be identified. The anastomotic blood vessels (ANAST) extending from the rete mirabile (RM) to the reformed internal carotid artery (IC) are demonstrated. The stump of the origin of the vestigial internal carotid artery (IC‐S) is identified. The tortuous course of the ciliary artery (CI) within the external rete is demonstrated. The position of the maxillary artery (MA) is in the inferior section of the rete mirabile (RM). Arterial abbreviations: AC, anterior cerebral; AP, ascending pharyngeal; BA, basilar; CC, common carotid; EC, external carotid; EE, external ethmoid; FA, facial; GP, greater palatine; IA, inferior alveolar; IC, internal carotid; IE, internal ethmoid; IO, infraorbital; LI, lingual; MC, middle cerebral; OC, occipital; PA, posterior auricular; PC, posterior cerebral; SC, superior cerebellar; ST, superficial temporal; VA, vertebral.

**Figure 3:**
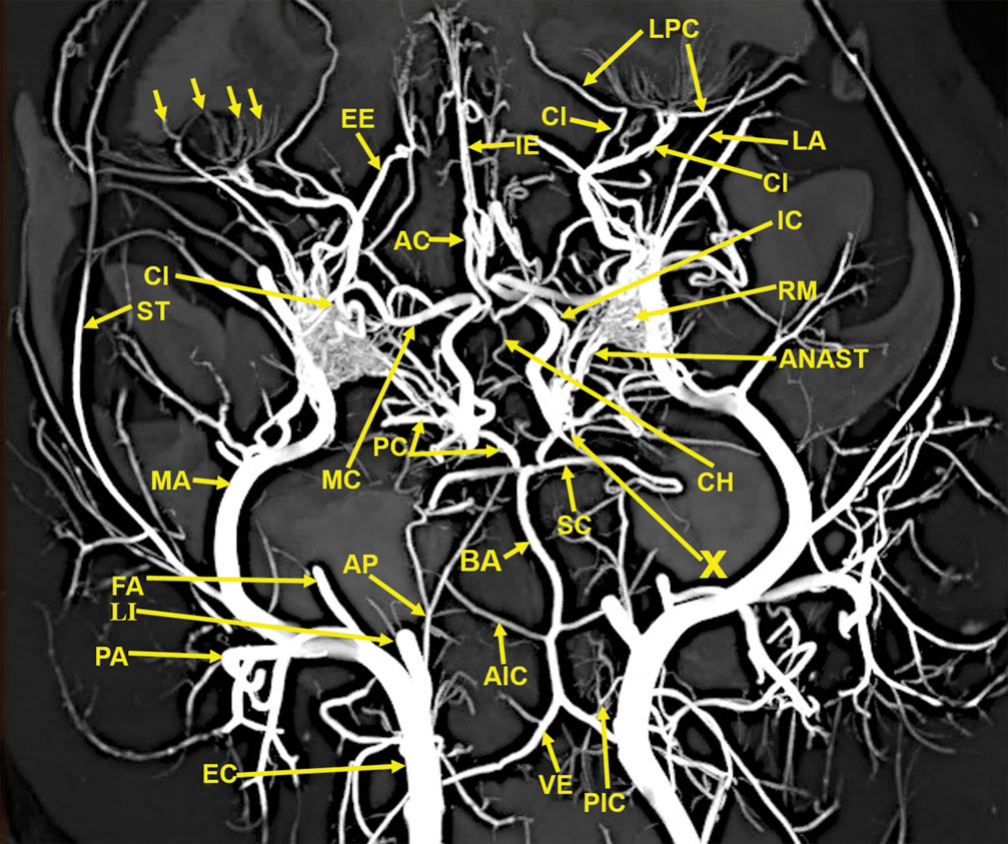
A 20‐mm‐thick oblique dorsoventral multiplanar reconstruction of the cranial arterial circulation of the cat, using maximum intensity projection. Among the many identified vascular structures are (1) The rete mirabile (RM). (2) The junction (X) of the multiple anastomotic vessels (ANAST) from the medial edge of the rete mirabile (RM), forming the internal carotid artery (IC). (3) The circle of Willis formed by internal carotid (IC), posterior cerebral/posterior communicating arteries (PC), and a small segment of the anterior cerebral (AC) arteries. (4) The origin of the external ethmoidal (EE) arteries within the rete (RM) is demonstrated. (5) The tortuous course of the ciliary artery (CI) within the rete (RM) also is demonstrated. (6) The long posterior ciliary artery (LPC) and the faint outline of the ciliary circle branches (small arrows) can be identified. (7) The position of the maxillary artery (MA) is in the inferolateral section of the rete mirabile (RM). Arterial abbreviations: AIC, anterior inferior; AP, ascending pharyngeal; BA, basilar; CH, chiasmatic; EC, external carotid; FA, facial; IE, internal ethmoid; LA, lacrimal; LI, lingual; PA, posterior auricular; PIC, posterior inferior; SC, superior cerebellar; ST, superficial temporal; VE, vertebral.

**Figure 4:**
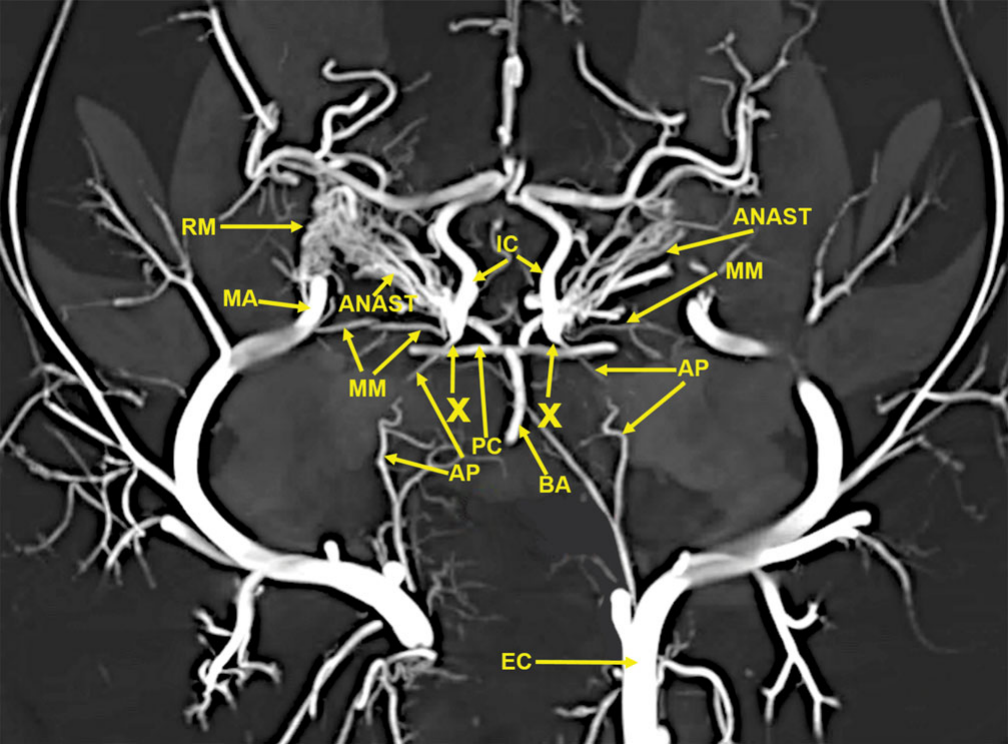
A 10‐mm‐thick oblique dorsoventral multiplanar reconstruction of the cranial arterial circulation of the cat, using maximum intensity projection. This thin slab shows the maxillary artery (MA) coursing within the rete (RM). The anastomotic blood vessels (ANAST) originating from the medial edge of the rete mirabile are coursing toward the origin of the reconstituted and functional internal carotid artery (IC). The ascending pharyngeal artery (AP) and the middle meningeal artery (MM) also have an important role in forming the reconstituted internal carotid artery. The region (X) is where the anastomotic blood vessels from the external rete, the ascending pharyngeal artery (AP), and the middle meningeal artery join to form the reconstituted functional internal carotid artery (IC). Arterial abbreviation: EC, external carotid artery.

**Figure 5:**
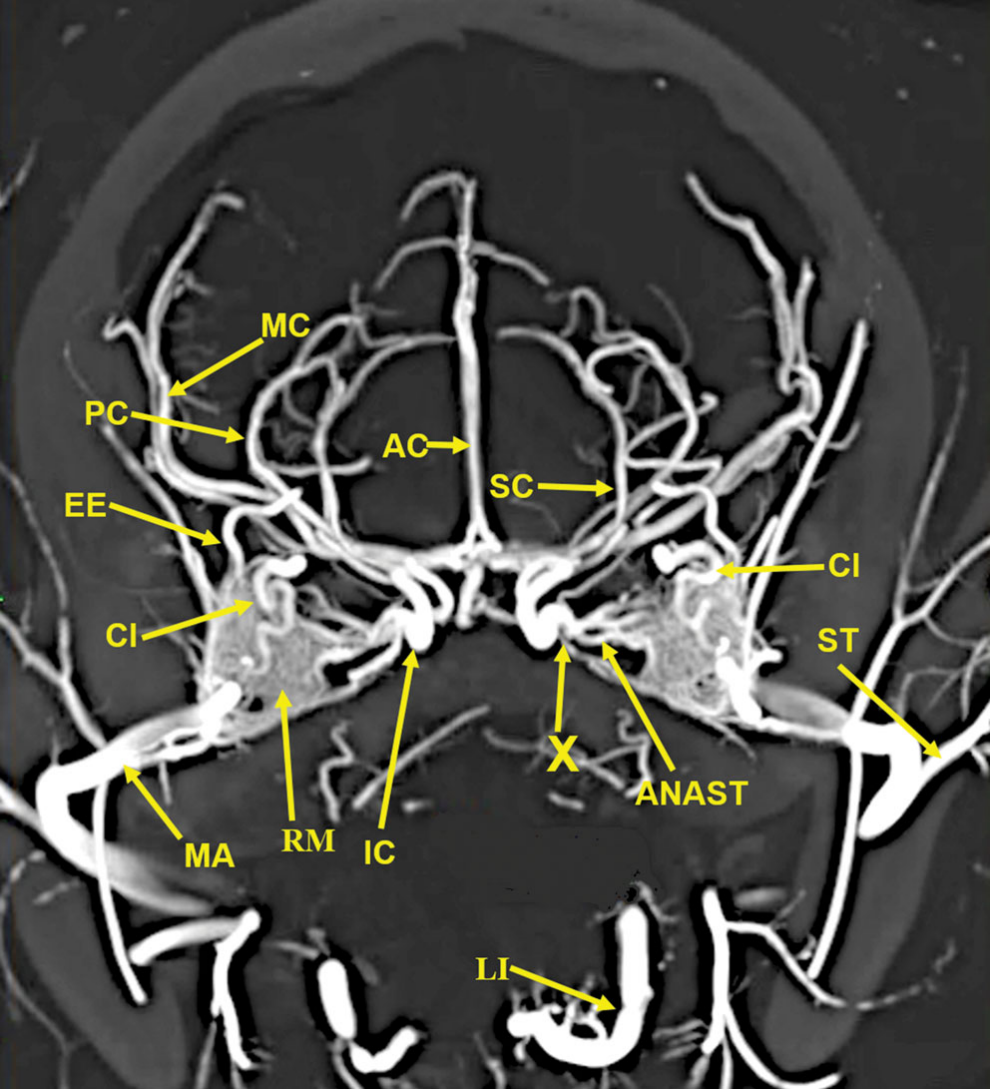
A 20‐mm‐thick anteroposterior multiplanar reconstruction of the cranial arterial circulation of the cat, using maximum intensity projection. Of note is the junction (X) of the multiple anastomotic vessels (ANAST) arising from the medial edge of the rete mirabile (RM), forming the internal carotid artery (IC). Also visible are the origin of the external ethmoid (EE) within the rete. The ciliary artery (CI), which has a tortuous course within the rete, is actually a branch of the internal maxillary artery (IM). Arterial abbreviations: AC, anterior cerebral; LI, lingual; MC, middle cerebral; PC, posterior cerebral; SC, superior cerebellar; ST, superficial temporal.

**Figure 6:**
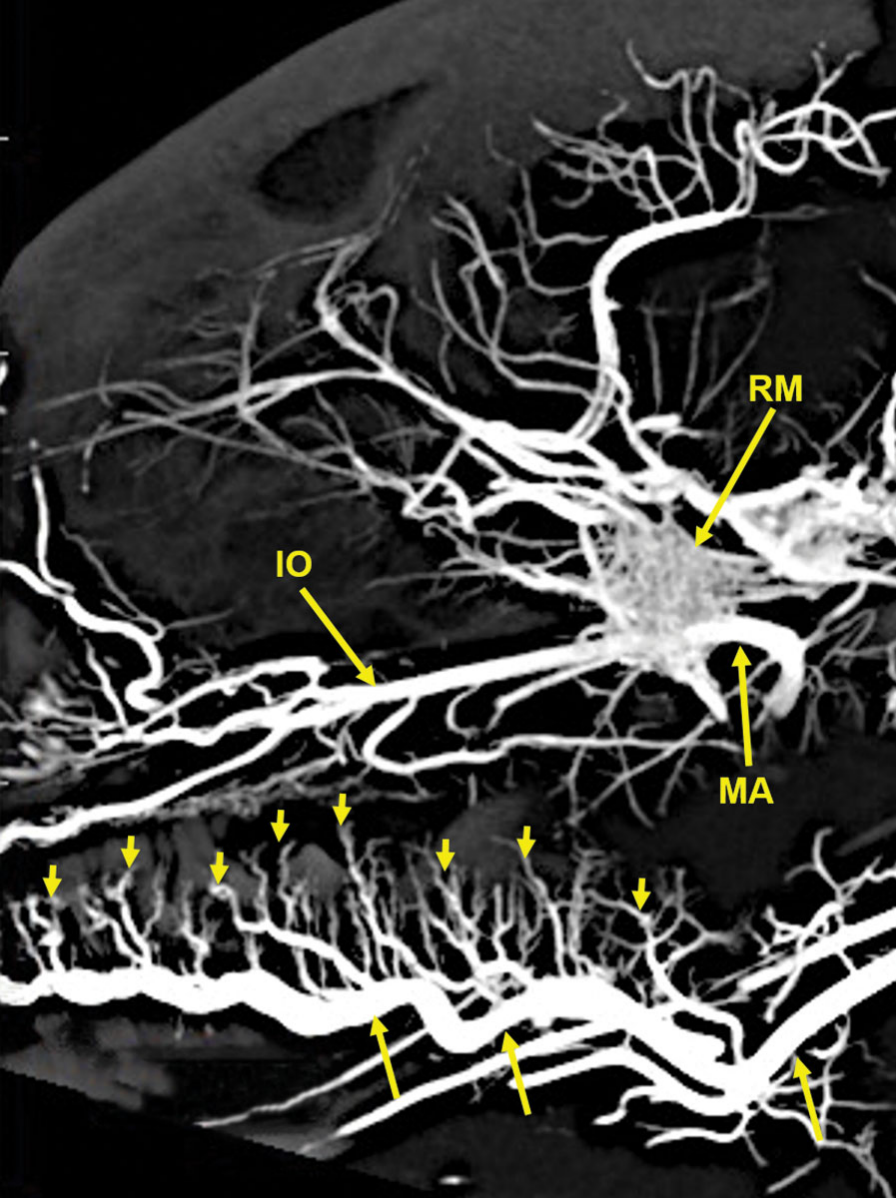
A 20‐mm‐thick lateral multiplanar reconstruction of the cranial arterial circulation of another cat, using maximum intensity projection. A large number of very small arteriolar branches (small arrows) of the lingual artery (longer arrows) are penetrating the tongue. Arterial abbreviations: IO, infraorbital; MA, maxillary; RM, rete mirabile.

**Figure 7:**
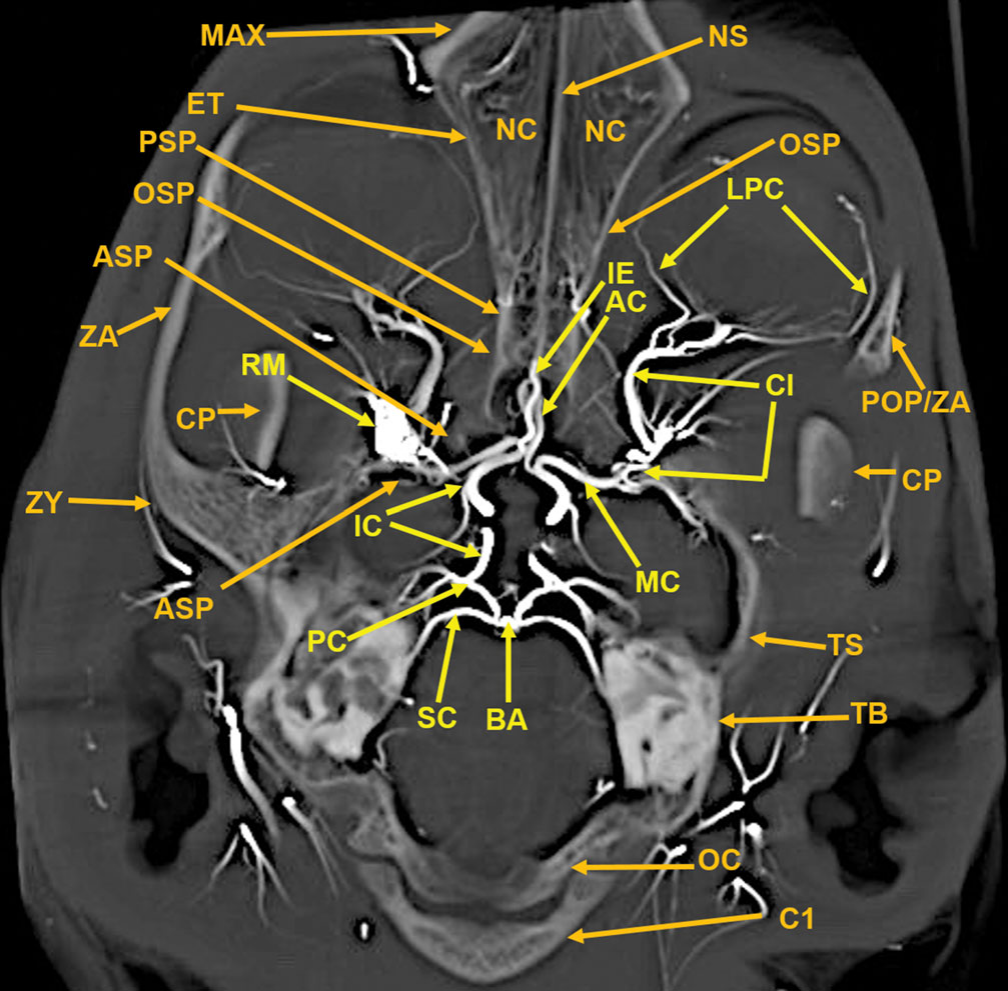
A 13‐mm‐thick dorsoventral multiplanar reconstruction of the cranium of the arterially injected cat using maximum intensity projection. The skull components are orange colored annotations; the arterial structures are in yellow color annotation in Figures 7, 8, 9, 10, 11, 12. The nasal cavity (NC) walls are the maxilla (MAX), ethmoid (ET), and orbitosphenoid (OSP) bones. The rete mirabile (RM) and its component, the tortuous ciliary artery (CL), pass through the orbital fissure, and the alisphenoid (ASP). Also identified are segments of the following components of the skull: C1, posterior arch of first cervical vertebra; CP, coronoid process; OC, occipital; POP/ZA, post orbital process of the zygomatic arch; PSP, presphenoid; TB, tympanic bulla; ZA, zygomatic arch; ZY, zygoma; and the following arteries: AC, anterior cerebral; BA, basilar; IC, internal carotid; IE, internal ethmoid; MC, middle cerebral; PC, posterior cerebral; SC, superior cerebellar.

**Figure 8:**
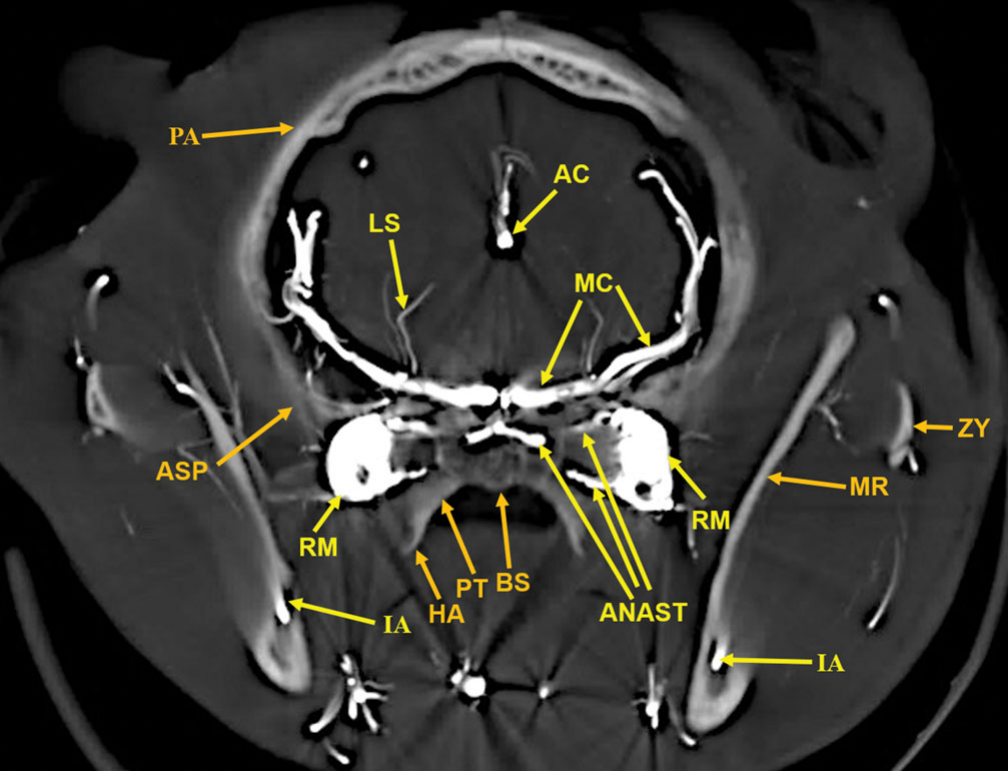
A 6‐mm‐thick anteroposterior multiplanar reconstruction of the cranium of the arterially injected cat using maximum intensity projection. The middle cerebral (MC) artery branches, the anterior cerebral (AC) artery branches, within the interhemispheric fissure, and the lenticulostriate (LS) arteries of the cerebrum are encased superiorly and laterally by the parietal (PA) bones and inferiorly by the alisphenoid (ASP) bones. The rete mirabile (RM) is situated lateral to the pterygoid bone (PT) with its inferior extension; the hamulus (HA). The pterygoid bone is fused medially with the basisphenoid (BS). Anastomotic (ANAST) arterial branches extend medially to participate in the reconstitution of the internal carotid artery at the circle of Willis as seen in Figure 5. Segments of the zygomatic arch (ZY) and of the mandibular ramus (MR) including the inferior alveolar artery (IA) are demonstrated.

**Figure 9:**
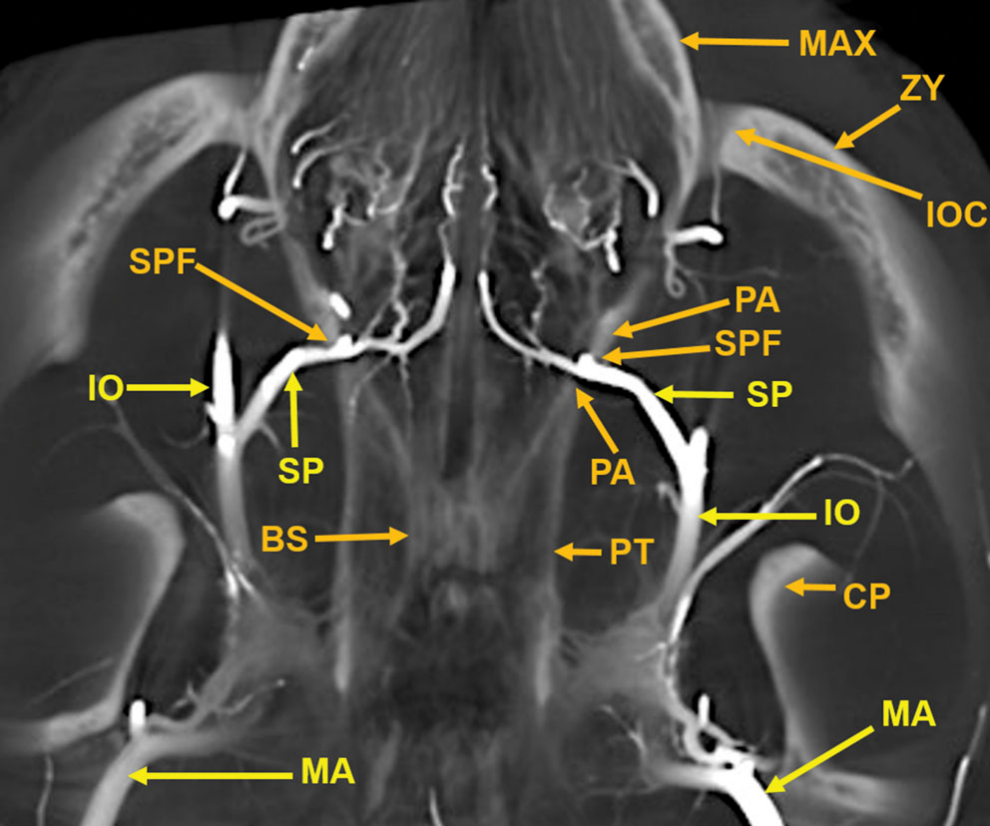
An 11‐mm‐thick dorsoventral multiplanar reconstruction of the cranium of the arterially injected cat using maximum intensity projection. The sphenopalatine (SP) arteries pass through the sphenopalatine foramina (SPF) to enter the nasal cavities. The sphenopalatine foramen is within the orbital process of the palatine (PA) bone. The vascular pattern in the nasal cavity is demonstrated in Figure 10. The sphenopalatine arteries are branches of the infraorbital (IO) arteries, which are the continuation of the maxillary (MA) arteries. The infraorbital artery continues anteriorly to pass through the infraorbital canal (IOC), which is within the zygoma (ZY) at the junction with the maxilla (MAX). The entire infraorbital canal with the infraorbital artery within it is seen in Figure 10. Also identified are segments of the bony basisphenoid (BS), the pterygoid (PT) bone, and the coronoid process (CP) of the mandible.

**Figure 10:**
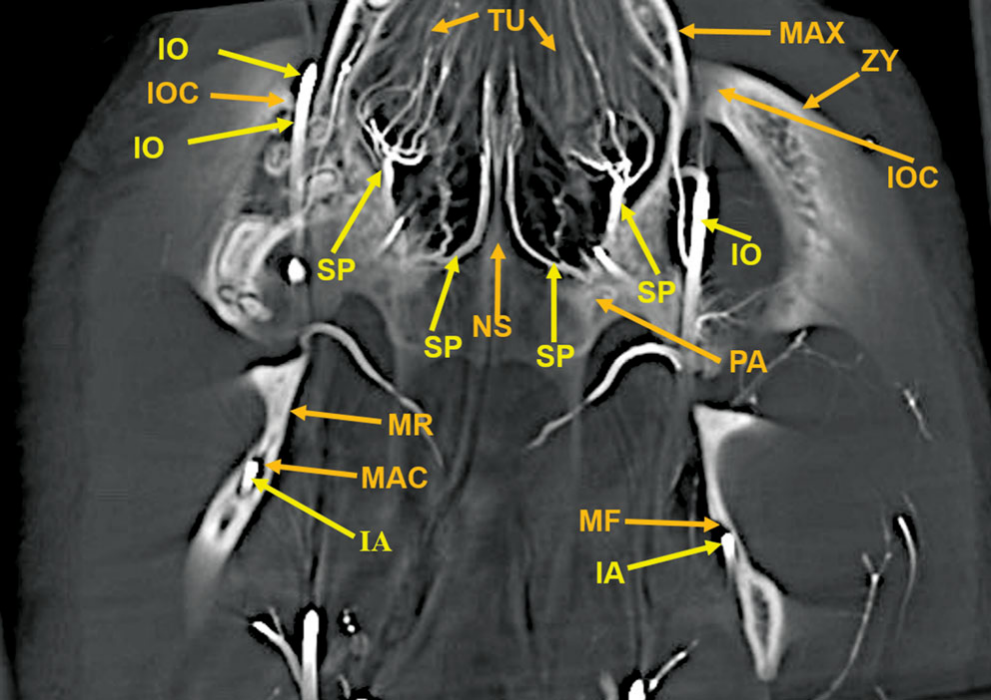
An 8‐mm‐thick dorsoventral multiplanar reconstruction of the cranium of the arterially injected cat using maximum intensity projection. The infraorbital artery (IO) passes through the infraorbital canal (IOC), within the zygoma (ZY). The latter is attached to the maxilla (MAX), forming the wall of the nasal cavity. The turbinates (TU) within the nasal cavity are supplied by branches of the sphenopalatine (SP) arteries which also surround the nasal septum (NS). The palatine (PA) bone adjacent to the sphenopalatine artery is identified. The inferior alveolar artery (IA) is within the mandibular canal (MAC) in the left mandibular ramus (MR) and in the mandibular foramen (MF) of the right mandibular ramus.

**Figure 11:**
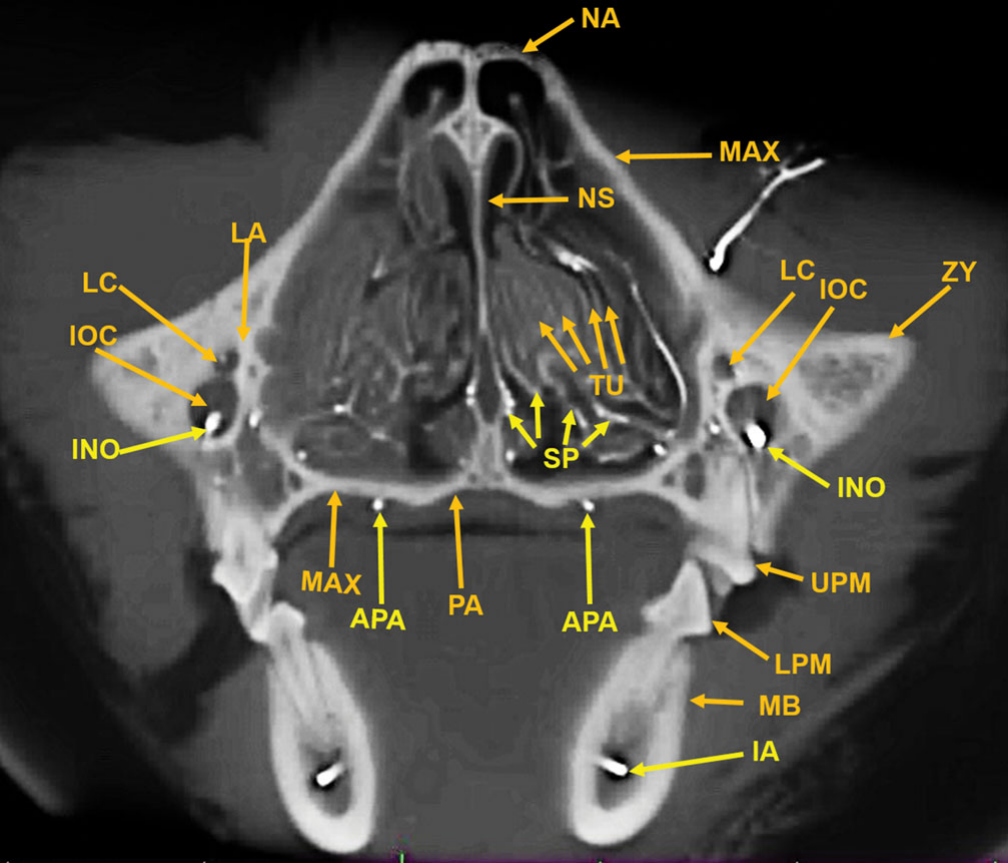
An 11‐mm‐thick anteroposterior multiplanar reconstruction of the cranium of the arterially injected cat using maximum intensity projection. The nasal (NA) bones form the roof of the nasal cavity. The bony nasal septum (NS) is attached at the suture between the two nasal bones. The lateral wall of the nasal cavity is formed by the maxilla (MAX), the lacrimal (LA) bone, and the segment of the zygoma (ZY) medial to the infraorbital canal (IOC). There is an extensive number of turbinates (TU) within the nasal cavity which are supplied by branches of the sphenopalatine (SP) arteries. The infraorbital artery (IO) passing through the infraorbital canal is identified with the smaller lacrimal canal (LC), for the lacrimal duct, adjacent to the infraorbital canal. The bony palate, the floor of the nasal cavity, at this level is formed laterally by the maxilla (MAX) and medially by the palatine (PA) bone. Also identified are the anterior palatine arteries (AP) below the palate; the inferior alveolar artery (IA) within the mandibular canal (MAC) in the mandibular body (MB) and the upper (UPM) and lower (LPM) premolar teeth.

**Figure 12:**
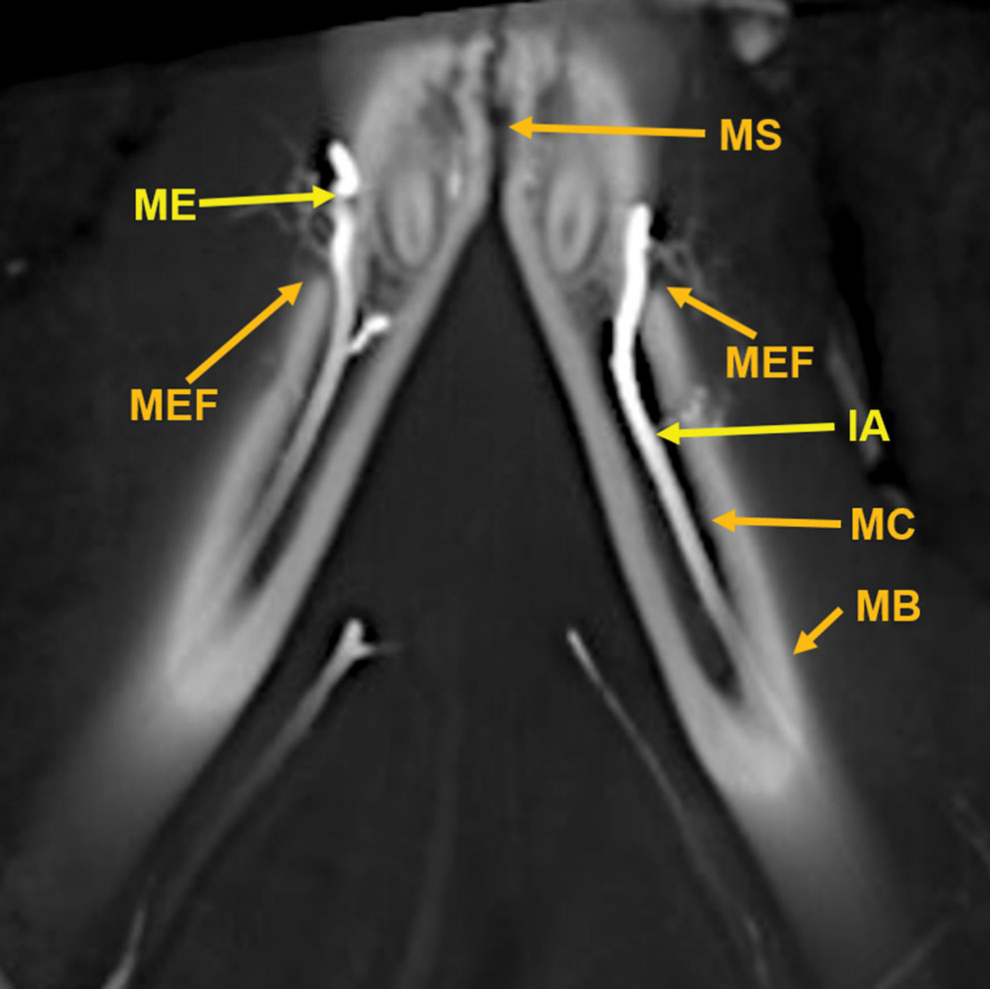
An 11‐mm‐thick dorsoventral multiplanar reconstruction of the mandible of the arterially injected cat using maximum intensity projection. Visualized are inferior alveolar (IA) arteries within the mandibular canals (MC) of the mandible (MB). The inferior alveolar arteries exit the mental foramina (MEF) becoming the mental (ME) arteries. The mandibular symphysis (MS) is visible.

The following arteries were identified: AC, anterior cerebral; AIC, anterior inferior; ANAST, anastomotic; AP, ascending pharyngeal; APA, anterior palatine; BA, basilar; CC, common carotid; CH, chiasmatic; EC, external carotid; EE, external ethmoid; FA, facial; GP, greater palatine; IA, inferior alveolar; IC, internal carotid; IE, internal ethmoid; IO, infraorbital; LA, lacrimal; LI, lingual; LPC, long posterior ciliary; LS, lenticulostriate; MA, maxillary; MC, middle cerebral; ME, mental; MM, middle meningeal; OC, occipital; PA, posterior auricular; PC, posterior cerebral; PIC, posterior inferior; SC, superior cerebellar; SP, sphenopalatine; ST, superficial temporal; and VE, vertebral.

Components of the following bones of the skull were identified: C1, posterior arch of first cervical vertebra; CP, coronoid process; ET, ethmoid; IOC, infraorbital canal; LA, lacrimal bone; MB, mandibular body; MAC, mandibular canal; MAX, maxilla; MB, mandible; MC, mandibular canal; MF, mandibular foramen; MS, mandibular symphysis; MR, mandibular ramus; MEF, mental foramen; NA, nasal bones; NS, nasal septum; OSP, orbitosphenoid; OC, occipital; POP/ZA, post‐orbital process of the zygomatic arch; PSP, presphenoid; TU, turbinates; TB, tympanic bulla; TS, temporal squama; ZA, zygomatic arch; and ZY, zygoma.

- The stump of the occluded nonfunctional vestigial internal carotid artery is present in Figure 2.
- The reconstitution of a functional internal carotid artery by several anastomotic blood vessels arising from the medial margin of the rete mirabile with a branch of the ascending pharyngeal artery and the middle meningeal artery was demonstrated (Figs. 3, 4, 5).
- The inferolateral position of the maxillary artery within the rete (Figs. 2, 3, 4, 5, 6).
- Visualization of the anastomotic arteries and the rete in several projections (Figs. 2, 3, 4, 5, 6, 7, 8).
- Visualization of the components of the sphenoid and pterygoid bones in the region of the rete (Figs. 7 and 8).
- The origin of the ciliary artery within the rete and its course toward the eye was shown in Figures 3 and 5.
- The visualization of the ciliary artery, long posterior ciliary artery, its branches, and the iris ring (Fig. 3).
- The identification of external and internal ethmoidal arteries (Figs. 2 and 3).
- The detailed vascular supply of the tongue was demonstrated in Figure 6.
- The identification of the infraorbital artery passing through the infraorbital canal (Figs. 9 and 10).
- The identification of the lacrimal canal (Fig. 10).
- The identification of the sphenopalatine arteries supplying the nasal turbinates (Figs. 9 and 10).
- The identification of the inferior alveolar artery, a branch of the internal maxillary at the level of the mandibular condyle, enters the mandibular canal to supply the mandibular teeth (Figs. 8, 9, 10).
- The identification of the sphenopalatine foramen (Fig. 9) and mental foramen (Fig. 12).

## DISCUSSION

The rete mirabile (Latin for “wonderful net”; plural‐“retia mirablia”) is a subgroup of a complex structure of arteries and veins with very thin walls using countercurrent blood flow resulting in exchange of heat (Scholander, 1957; Williams et al., 1989), gasses (Scholander, 1957), and ions (Scholander, 1957; Williams et al., 1989) in different organs of the body. The rete mirabile has also been variously named in the literature as the carotid rete, epidural rete, and extracranial rete. The term rostral epidural rete mirabile has also been used (Frąckowiak and Jakubowsk, 2008) as defined in Nomina Anatomica Veterinaria (5th ed., 2012, p. 77).

A fully developed rete has been described in the cat, as well as in the sheep, goat (*Capra hircus*), ox (*Bos taurus*), and pig (*Sus scrofa*), and other artiodactyls, with a rudimentary one in the dog (*Canis familiaris*) (Gillilan, 1974; du Boulay et al., 1975; Gillilan, 1976; Strauss et al., 2017; O'Brien, 2018a, 2018b). There is no rete in the rabbit (*Oryctolagus cuniculus*) and rat (*Rattus norvegicus*) (Daniel et al., 1953). In felids such as the cat, leopard (*Panthera pardus*), ocelots (*Leopardus pardalis*), tiger (*Panthera tigris*), as well in even‐toed ungulates, the internal carotid artery is not the source of blood supply to the brain (Gillilan, 1976). This is also the case in the lion (*Panthera leo*) (Hsieh and Takemura, 1994; Frąckowiak and Godynicki, 2003). Studies of the cat, lion, leopard, tiger, serval (*Leptailirus serval*), lynx (*Lynx lynx*), leopard cat (*Felis begalensis*), jungle cat (*Felis chaus*), puma (*Felis concolor*), and jaguar (*Pathera onca*) also demonstrated that the internal carotid artery was not the source of blood to the brain (Frąckowiak and Godynicki, 2003; Frąckowiak and Jakubowsk, 2008). Instead, the external carotid artery via the maxillary artery supplies the brain via a highly specialized vascular plexus named the rete mirabile. In the cat, it is located extracranially, in the pterygoid fossa. In other species, it is located intracranially, within the cavernous sinus (Daniel et al., 1953; Frąckowiak and Godynicki, 2003).

Several casting methods of the retia in mammals, including the cat, have been described in the literature. Casting was described with latex (Davis and Story, 1943), neoprene latex (Daniel et al., 1953), colored methyl methacrylate (Gillilan and Markesbery, 1963), India ink and gelatin (Martinez, 1965), plastoid® resin (Martinez, 1967), vinylite (McFarland et al., 1979), acryl plastic (Takemura, 1982), vinyl superchloride, Technovit 7001 or Latex LBS 3060 (Frąckowiak and Godynicki, 2003; Frąckowiak and Jakubowsk, 2008) latex and stained vinyl superchloride dissolved in acetone (Frąckowiak and Jakubowsk, 2008), pigmented acetone solution of vinyl superchloride (Kiełtyka‐Kurc et al., 2015), and Liquid Polibar Plus barium sulfate suspension in red liquid latex (O'Brien, 2015; O'Brien and Bourke, 2015; O'Brien et al., 2016). The casts have been described in great detail in some of these publications.

Very detailed colored diagrammatic description of the rete of the cat was done by Davis and Story (1943) and reproduced in a textbook of cat anatomy (Crouch, 1969). Also available are very detailed black and white diagrams of the cat rete mirabile (Martinez, 1965; Takemura, 1982). A single detailed plain radiographic image of the cat's carotid rete was obtained by an intravascular injection of a colloidal micropaque mixed with carbon black (Kamijyo and Garcia, 1975).

In the cat, the common carotid artery, at the level of the thyroid cartilage, gives off three branches: the internal carotid, the occipital, and the ascending pharyngeal arteries (Fig. 2). The internal carotid, although of normal size in the fetal cat (Davis and Story, 1943), becomes progressively vestigial in the postnatal period, starting at its origin from the common carotid artery where it enters the skull through a tiny carotid foramen next to the lateral foramen lacerum. The internal carotid artery only becomes functional after the ascending pharyngeal artery enters the cavernous sinus where it joins with the large anastomotic branches from the rete, and a branch of the middle meningeal artery, to reconstitute the internal carotid artery at the level of the circle of Willis (Figs. 3, 4, 5). The evolutionary relationship of the internal carotid and the ascending pharyngeal arteries and the possible substitution by the ascending pharyngeal for a section of the vestigial internal carotid artery have been described, including a diagrammatic illustration by Martinez (Davis and Story, 1943; Martinez, 1965; du Boulay et al., 1975). In the dolphin (*Tursiops truncatus*) the embryonic carotid and vertebral arterial system regress and are replaced by a massive thoracico‐spinal and cranial rete system (McFarland et al., 1979). These authors in their extensive presentation (McFarland et al., 1979) point out that in phylogeny, there is progression from brains supplied only by the carotid system to the mammalian brains which are supplied both by the carotid and vertebral systems. A recent study points out that the amount of obliteration of the internal carotid artery, and the proliferation of the rete mirabile are quite variable among various even‐toed ungulates (O'Brien et al., 2016). This may be a factor in the thermoregulatory adaption of the various angulates. Recent studies have shown that selective brain cooling functions mainly as water‐conserving mechanism allowing certain artiodactyls to conserve more than half of their daily water requirements (Strauss et al., 2017). Thus, the artiodactyls may have selective advantage in the drier and hotter conditions of climate change.

Some authors name the region where the anastomotic vessels from the extracranial rete (in he pterygoid fossa) join with a branch of the ascending pharyngeal artery, to reconstitute a functional internal carotid artery at the level of the circle of Willis, as the internal rete (Davis and Story, 1943; Kamijyo and Garcia, 1975). Other authors have not found an internal rete (Daniel et al., 1953; Takemura, 1982). The region in dispute and named the internal rete is indicated with an arrow named X in Figures 3, 4, 5. The rete in the pterygoid fossa is a complex of very small vessels intertwined with the venous pterygoid plexus that completely surrounds the maxillary artery (Davis and Story, 1943; Martinez, 1967). Histologic studies of the external rete show a plexus of multiple very small, thin‐walled vessels with multiple arteriovenous anastomoses surrounding the maxillary artery. The rete consists of tightly packed and interlaced tiny vessels that supply the circle of Willis, the orbit, and the ethmoidal region of the nose from the external carotid artery via the maxillary artery and the extracranial rete (Daniel et al., 1953).

This study shows the origin of the ciliary and external ethmoidal arteries within the lateral rete (Figs. 2, 3, and 5). Daniel et al. (1953) describe the external ethmoidal artery as originating in the rete. However, the ciliary artery, which has a tortuous course within the rete, does not originate within the rete. It is actually a branch of the maxillary artery (Daniel et al., 1953). This is also seen in Figure 5 showing the tortuous ciliary, within the rete, is adjacent to a dense segment of the maxillary artery.

A comprehensive study of the rete of the cat (Takemura, 1982) describes the following afferent arterial vessels of the rete: the posterior deep temporal, inferior alveolar, middle meningeal, maxillary, buccal, anterior deep temporal, and zygomatic arteries. The following efferent vessels were observed: external ethmoidal, meningeal, extraocular muscular, lacrimal, a branch communicating with the external ethmoidal, temporal, interretial, and the anastomotic arteries. The anastomotic arteries surrounding the maxillary artery within the center of the rete are augmented by inflow from the surrounding layers of many afferent arteries and become the largest efferent arteries and a major component of cerebral blood supply to the brain in place of the vestigial internal carotid (Takemura, 1982).

For many years, there has been continuing research regarding the physiologic role of the rete mirabile, among them is the cooling of the hypothalamic region by venous blood flow from the nose and mouth (Baker, 1972; du Boulay et al., 1975). The possible role of the rete in the autoregulation of vasoconstriction and blood pressure in the cat and even‐toed ungulates has been reviewed (Daniel et al., 1953; du Boulay and Darling, 1975). In a study of the dolphin, another possible role of the rete is postulated to be the pressure‐damping effect of the rete as demonstrated by the nonpulsatile pressure profile in the efferent retial arteries (Nagel et al., 1968). This may be related to the need for rapid redistribution of blood in various organs in diving mammals.

Recent publications have extensively reviewed these topics, focusing on the various physiological roles of the carotid rete, including water conservation through selective brain cooling mechanisms and the history of multiple different evolutionary modifications (O'Brien, 2015; Strauss et al., 2017; O'Brien, 2018a, 2018b). Although these authors focus on even‐toed ungulates, they note that the rete mirabile was first studied in the cat. They note that water is conserved, and the hypothalamic region is selectively cooled by the cooler venous blood from the nasal mucosa and other facial veins passing through the complex architecture of the rete, lowering the arterial blood temperature in the hypothalamic region, thus also benefitting predators such as the cat.

## CONCLUSIONS

The obtained images demonstrated, with a nondestructive method, the high‐resolution vascular anatomy of the cerebral, orbital, facial arterial system, the rete mirabile, and skull bone components of the cat, with details not previously described in the literature. The images also demonstrated the durability of the injected contrast medium. This investigation also pointed out the scientific value of preserving specimens for prolonged periods until imaging techniques have made major advances in providing microanatomic detail in large specimens.

## CONFLICT OF INTEREST

The authors declare no competing or financial interest.

## AUTHOR CONTRIBUTIONS

E. Leon Kier contributed the concept/design, created and analyzed the various sets of OsiriX MD images, annotated the figures, and wrote the draft, final version, and revisions of the manuscript. G. J. Conlogue developed and produced the barium–gelatin contrast medium, performed the preparations, injections, and preservation of the specimens, and reviewed the draft, the final version, and revision of the manuscript. Z. Zhuang performed the scans of the specimens on the GE CT120 μCT high‐resolution small‐bore scanner, created the DICOM data sets, established and described the scanning parameters, and reviewed the final version and revision of the manuscript.
